# Chemogenetics identifies separate area 25 brain circuits involved in anhedonia and anxiety in marmosets

**DOI:** 10.1126/scitranslmed.ade1779

**Published:** 2023-04-05

**Authors:** Christian M. Wood, Laith Alexander, Johan Alsiö, Andrea M. Santangelo, Lauren McIver, Gemma J. Cockcroft, Angela C. Roberts

**Affiliations:** 1 Department of Physiology, Development and Neuroscience, University of Cambridge, Cambridge, United Kingdom; 2 Behavioural and Clinical Neurosciences Institute, University of Cambridge, Cambridge, United Kingdom; 3 Department of Psychology, University of Cambridge; Cambridge, United Kingdom; 4 Professorial Fellow, Girton College, University of Cambridge, Huntington Road, Girton, Cambridge, CB3 0JG

## Abstract

Poor outcomes are common in individuals with anxiety and depression, and the brain circuits underlying symptoms and treatment responses remain elusive. To elucidate these neural circuits, experimental studies must specifically manipulate them, which is only possible in animals. Here, we used a chemogenetics strategy involving engineered designer receptors exclusively activated by designer drugs (DREADDs) to activate a region of the marmoset brain that is dysfunctional in human patients with major depressive disorder, called the subcallosal anterior cingulate area 25 (scACC-25). Using this DREADDs system, we identified separate scACC-25 neural circuits that underlie specific components of anhedonia and anxiety in marmosets. Activation of the neural pathway connecting the scACC-25 to the nucleus accumbens (NAc) caused blunting of anticipatory arousal (a form of anhedonia) in marmosets in response to a reward-associated conditioned stimulus in an appetitive Pavlovian discrimination test. Separately, activation of the circuit between the scACC-25 and the amygdala increased a measure of anxiety (the threat response score) when marmosets were presented with an uncertain threat (human intruder test). Using the anhedonia data, we then showed that the fast-acting antidepressant ketamine when infused into the NAc of marmosets prevented anhedonia after scACC-25 activation for more than one week. These neurobiological findings provide targets that could contribute to the development of new treatment strategies.

## Introduction

Chemogenetics strategies, for example where receptors are engineered to respond to small molecules, have promise for revealing symptom-specific neural circuit dysfunction in psychiatric disorders. However, their potential has not been realized in non-human primate models thus far. To gain insight into the causal neurobiology underlying neuropsychiatric symptoms, such as anxiety and anhedonia (the loss of pleasure), these strategies need to be applied in non-human primates in which the overall structural organization of the brain is more similar to humans than that of rodents (1, 2).

Symptoms such as anxiety and anhedonia have multiple neurobiological and psychological causes (3, 4). Indeed, as we have already shown, multiple regions of prefrontal and cingulate cortex in the primate brain may contribute differentially to anxiety (1). Such differential neurobiological causes likely explain the variability in treating disease symptoms across the patient population. Experimental studies in non-human primates offer the opportunity to dissect out the specific contributions of specialized brain regions or pathways to disease symptoms and importantly to differentiate the efficacy of distinct classes of therapeutics.

The ventromedial prefrontal cortex (vmPFC) is a region consistently implicated in major depressive disorder (MDD). Not only is dysregulation within vmPFC associated with MDD symptoms, but it is also ameliorated following successful treatment. The vmPFC, however, is a heterogeneous region and contains multiple distinct areas (5) involved in emotion regulation, memory and decision making (reviewed in 6). In particular, area 25 within the subcallosal anterior cingulate cortex (scACC-25), is a promising target for neurobiologically-driven therapies such as deep brain stimulation (7), but its causal relationship with the hallmark symptoms of MDD, in particular anhedonia and comorbid anxiety, are unknown.

Recent studies in the common marmoset have provided evidence for the contribution of pharmacologically-induced scACC-25 overactivation in both blunted positive emotion and heightened negative emotion similar to that seen in patients with MDD (8, 9). They also revealed the differential sensitivity of anhedonia-like, but not anxiety-like, symptoms in marmosets to an acute, systemic dose of ketamine. Critical questions remain, however, especially with regard to what the underlying mechanisms are by which these distinct symptoms are generated and differentially ameliorated.

Viral-mediated interventions in a non-human primate brain can reveal these neural circuit-specific mechanisms, determining whether common or dissociable pathways from scACC-25 to other brain regions underlie the comorbidity of anhedonia and anxiety in MDD. Specifically, here we focused on the scACC-25 projections to the nucleus accumbens (NAc) and amygdala because both regions have been implicated in reward and punishment processing (10–13). Therefore, we aimed to assess the causal involvement of these scACC-25 pathways separately in changes in reward and threat responses in marmosets (3, 14).

## Results

### Selective activation of separate scACC-25 pathways using chemogenetics

**Fig 1:**
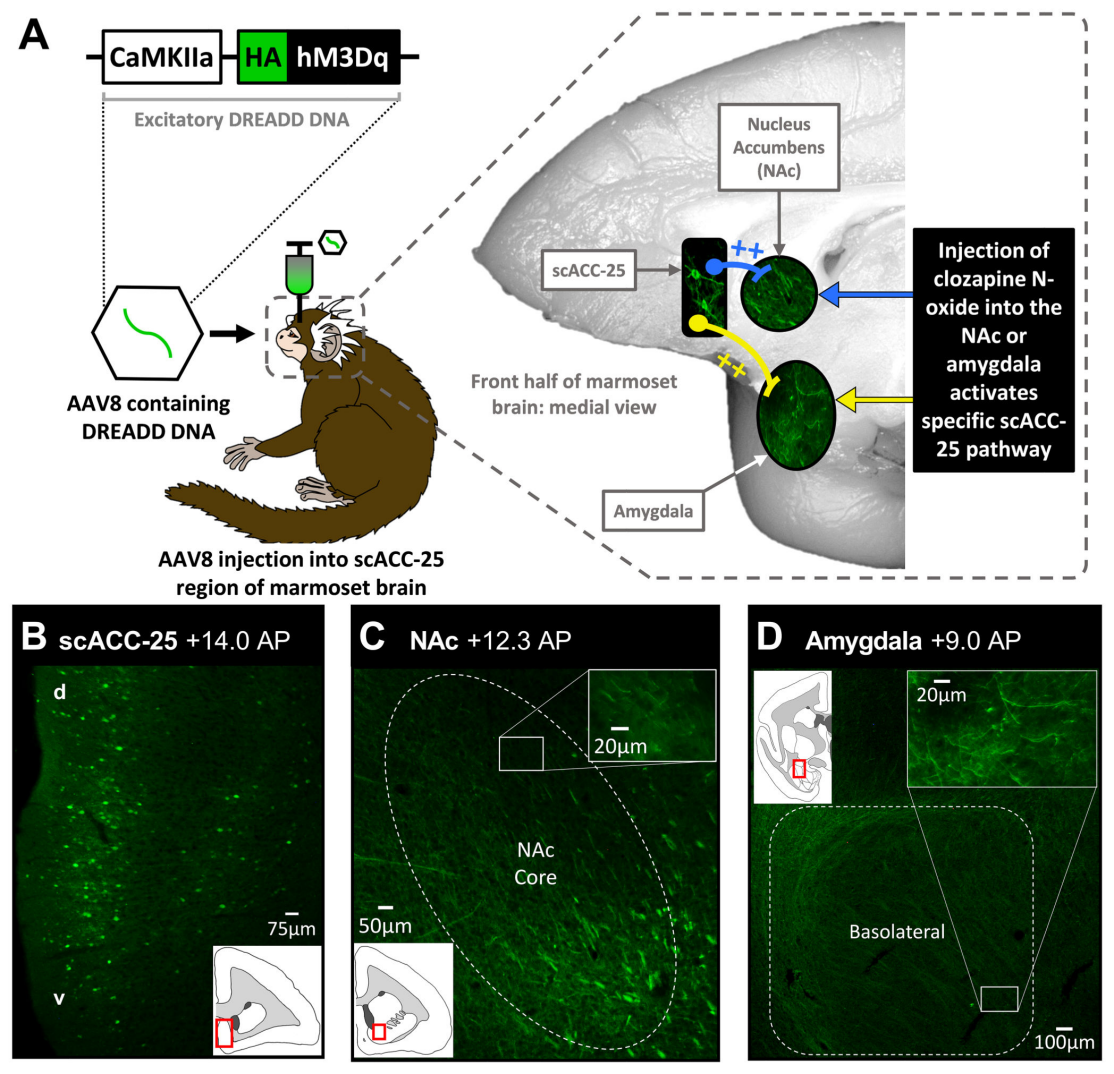
Activation of scACC-25 pathways in marmoset brain by the DREADD hM3Dq. (**A**) A DNA construct was created containing the excitatory DREADD hM3Dq fused to a hemagglutinin (HA) tag under control of a promoter (CaMKIIa) targeting primarily excitatory output neurons. This construct was packaged into an adeno-associated virus (AAV8) that was surgically injected into the scACC-25 of the marmoset brain. Inside the marmoset brain (grey dashed box), scACC-25 cell bodies expressed the DREADD protein as did their axons/terminals located in the nucleus accumbens (NAc) and amygdala. Infusion of a designer drug, such as clozapine N-oxide (CNO), into the NAc or amygdala specifically activated scACC-25-NAc (blue) and scACC-25-amygdala (yellow) pathways, respectively. The impact of activating these pathways could then be studied in the marmosets. (**B**) Photomicrograph of a coronal section through the subcallosal cingulate cortex of marmoset brain at a specific anteroposterior (AP) co-ordinate (+14.0) displaying anti-HA tag staining in scACC-25 cells. Widespread hM3Dq expression can be seen across dorsal (d) and ventral (v) aspects of scACC-25 (inset schematic shows location of target region). (**C, D**) Photomicrographs of coronal sections through the core subregion of (C) the NAc (NAc Core) and (D) the basolateral portion of the amygdala, illustrating fibers from hM3Dq expressing scACC-25 neurons (inset coronal sections show regions of interest).

We expressed the excitatory designer receptor exclusively activated by designer drugs (DREADD) hM3Dq, using an adeno-associated viral (AAV) vector under a calcium calmodulin kinase IIa (CaMKIIa) promoter, in scACC-25 of the marmoset brain (Fig. 1A), which primarily targets excitatory output neurons. Neurons in the scACC-25 of postmortem marmoset brain samples showed clear DREADD expression (Fig 1B, Fig S1). hM3Dq activation is known to induce changes in neuronal excitability and depolarization through intracellular Ca^2+^ release. Separate activation of the NAc and amygdala pathways from scACC-25 was achieved by infusion of DREADD activators, clozapine N-oxide (CNO) or deschloroclozapine, into the NAc or amygdala terminal regions through intracerebral cannulae (Fig 1C, D; Fig S2, S3).

### Anticipatory anhedonia and heightened responsivity to an uncertain threat are induced by two separate scACC-25 pathways

The effects of activating two separate scACC-25 pathways were determined by conducting two behavioral tests with the marmosets: (i) an appetitive Pavlovian discrimination test that measures reward anticipation and consumption, which can be blunted in anhedonia (15), and (ii) a human intruder test, which measures a marmoset’s anxiety-like responsivity to an uncertain threat in the form of an unknown human.

**Fig 2:**
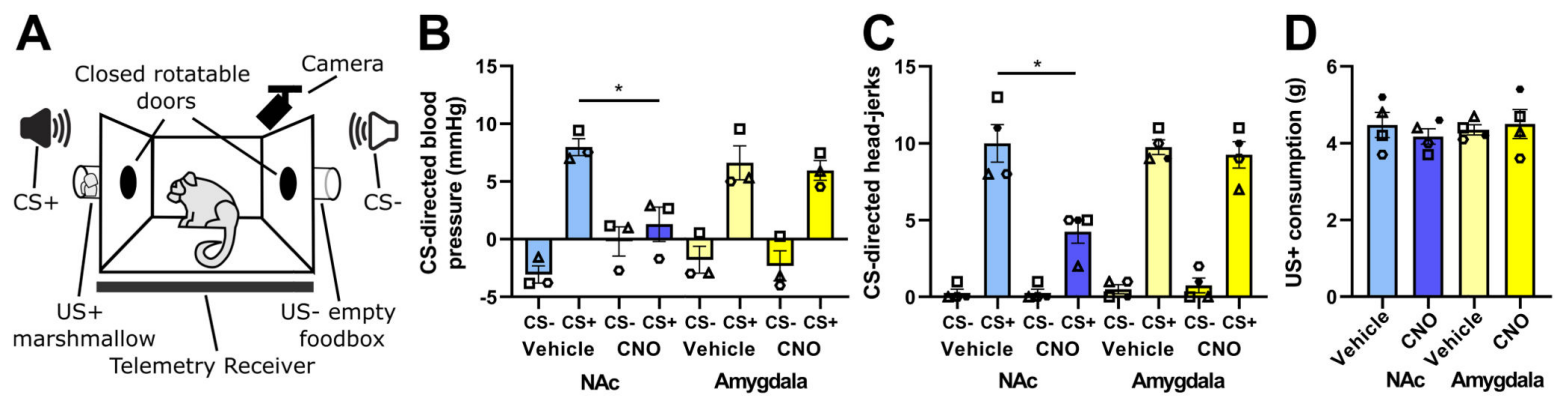
Projections from scACC-25 to NAc drive anticipatory anhedonia in marmosets. (**A**) Anticipatory and consummatory responses were studied in an appetitive Pavlovian discrimination test. This test uses two 20 second auditory cues, called the conditioned stimuli (CS), that were separately paired with either access to a food reward (CS+) or an empty food box (CS-). (The marshmallow reward is called the unconditioned stimulus; US+; empty food box, US-). (**B**) CNO infusion into the marmoset NAc (but not vehicle infusion) blunted blood pressure changes in response to the reward-associated conditioned stimulus (CS+). This was a pathway-specific effect as CNO infusion into the amygdala did not result in a significant difference in blood pressure in response to a reward-associated conditioned stimulus (CS+) compared to vehicle [n=3, three-way repeated measures (rm)ANOVA, treatment*region*CS interaction, F_(1,2)_=89.5, p=0.011, η=0.98; Sidak-corrected posthoc: CS+ NAc vehicle vs CNO, p=0.046, *d*=3.3; CS+ NAc CNO vs Amygdala CNO, p=0.038, *d*=2.2). (**C**) This was also observed for arousal in the form of head-jerk behaviors in response to a reward-associated conditioned stimulus (CS+). CNO infusion into the marmoset NAc (but not vehicle infusion) blunted CS+-directed head-jerks, whereas CNO infusion into the amygdala had no effect (n=4, three-way rmANOVA, treatment*region*CS, F_(1,3)_=11.538, p=0.043, η^2^=0.79; treatment*region, F_(1,3)_=14.520, p=0.032, η^2^=0.83; Sidak-corrected posthoc: CS+ NAc vehicle vs CNO, p=0.011, *d*=2.8 ; CS+ NAc CNO vs Amygdala CNO, p=0.001, *d*=3.1). (**D**) These effects were specific for reward anticipation as consumption of the reward was unaffected by CNO infusion in either region (n=4, two-way rmANOVA, treatment*region, F<1; treatment, F<1; region, F<1). Mean data are displayed with SEM error bars and individual data points are shown. Significant Sidak-corrected comparisons indicated by p<0.05*.

The appetitive Pavlovian discrimination test uses two 20 second auditory cues, called the conditioned stimuli, which are separately paired with either access to a food reward, the positive unconditioned stimulus (US+), or an empty food box, the negative unconditioned stimulus (US-), for 120 seconds (Fig 2A). Marmosets learn that one auditory cue predicts access to a reward, whereas the other does not. The animals show increases in their orienting behavior, known as head-jerking, and mean arterial blood pressure in response to only the reward-paired conditioned stimulus in anticipation of the reward but not the non-reward paired conditioned stimulus. The cardiovascular arousal mirrors the autonomic arousal commonly used to assess emotional responsivity in human patients with MDD (16). Importantly, this cardiovascular arousal is independent of general behavioral arousal, as it does not correlate with locomotor activity (Fig S4).

Infusion of CNO into the NAc resulted in activation of the scACC-25-to-NAc pathway and blunting of the anticipatory blood pressure response to the reward-associated conditioned stimulus (CS+), compared to infusion of a vehicle control into the same region (n=3, Fig 2B, blue and light blue bars respectively; p=0.046). This effect was selective for this pathway as infusion of CNO into the amygdala had no effect (yellow bars). Similarly, anticipatory head-jerk behaviors to the reward-associated conditioned stimulus (CS+) were reduced by selective activation of the scACC-25-to-NAc pathway (n=4, Fig 2C, p=0.011), with no effect after activation of the scACC-25-to-amygdala pathway. These effects were specific for reward anticipation because food reward consumption was unaffected, with the amount of food reward eaten remaining stable (Fig 2D). The blood pressure response to consumption of the marshmallow reward, the unconditioned stimulus (US+), remained unchanged (Fig S5). This specificity in affecting anticipatory rather than consummatory anhedonia recapitulates the symptoms experienced by MDD patients (3, 17). For additional confirmation, we used the alternative DREADD activator deschloroclozapine (100nM). When infused into the marmoset NAc, deschloroclozapine induced an identical blunting of anticipatory cardiovascular and behavioral arousal in response to the food reward-associated conditioned stimulus (Fig S6).

**Figure 3:**
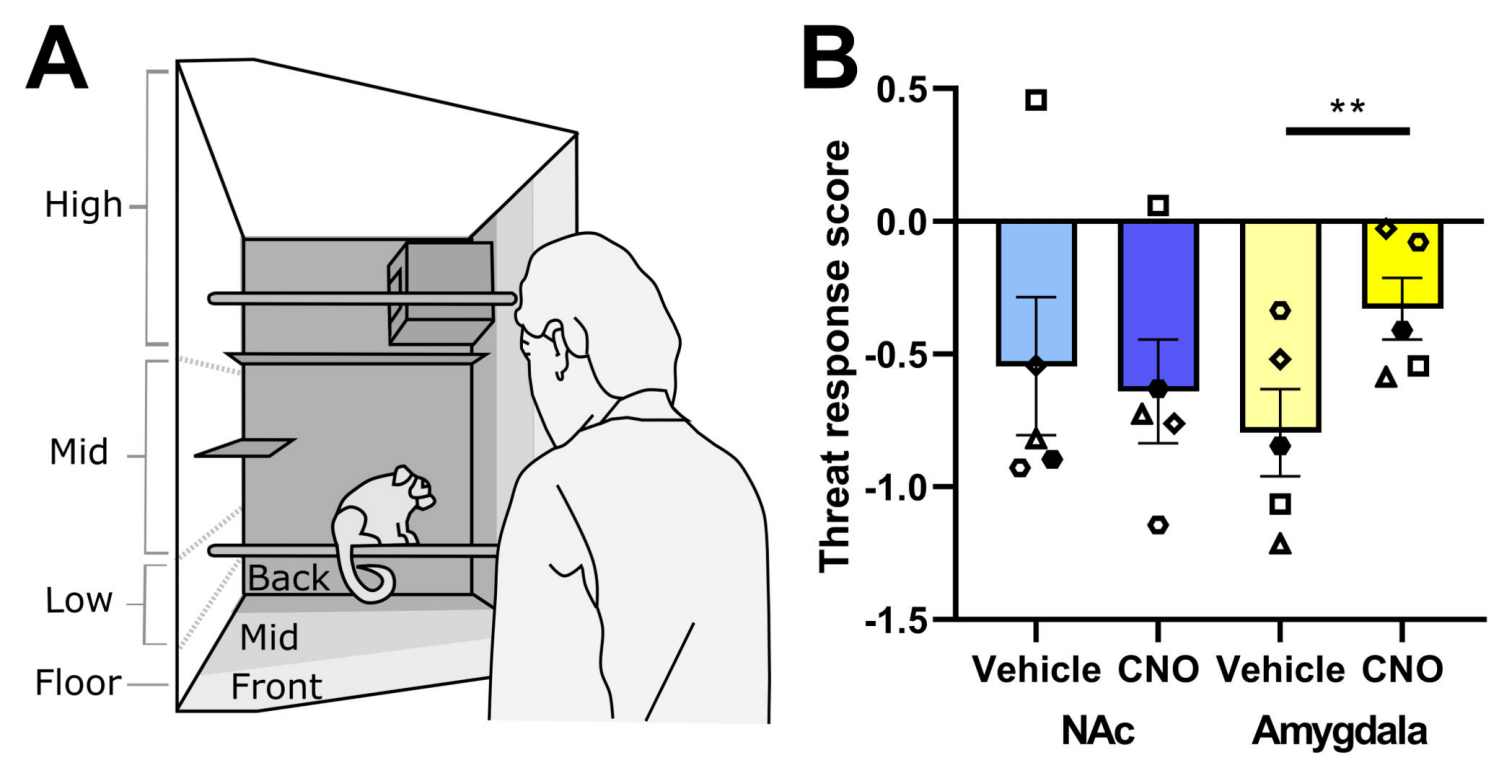
Projections from scACC-25 to the amygdala drive anxiety in marmosets. (**A**) Anxiety-like behaviors were analysed in the human intruder test, which assessed the marmoset’s response to an unknown human during a 2-minute test period. A single factor score reflecting intolerance of uncertain threat (the threat response score) was determined (Fig S7). (**B**) CNO infusion into the amygdala increased the threat response score in comparison to vehicle (yellow bars) in all marmosets, whereas CNO infusion into the NAc had no effect (blue bars) (n=5, two-way rmANOVA; treatment*region, F_(1,4)_=20.3, p=0.011, η^2^=0.84; Sidak-corrected posthoc, Amygdala vehicle vs CNO, p=0.002, *d*=1.5). Data from individual measures that contributed to the threat response score are provided in Table S1. Mean data are displayed with SEM error bars and individual data points are shown. Significant Sidak-corrected comparisons indicated by p<0.01**.

Next, we used the human intruder test to study responsivity to an uncertain threat, a component of anxiety. This test measures a marmoset’s response to an unknown human who maintains eye contact for two minutes (Fig 3A). The uncertainty is born from the fact that both positive and negative experiences have previously arisen from interactions with unknown humans. A rich behavioral repertoire, including species-specific responses are assessed in this test and are combined into a threat response score derived from an exploratory factor analysis of more than 170 marmosets (Fig S7).

Compared to reward anticipation, converse effects were seen in the human intruder test (n=5). Specifically, CNO infusion into the amygdala resulted in marmosets displaying heightened reactivity to the human intruder indicated by an increase in the threat response score compared to vehicle control infusion into the amygdala (Fig 3B; p=0.002). Although no specific individual variable drove this effect, in general, the marmosets spent less time at the front of the cage (Table S1) suggesting a higher level of avoidance and thus a greater threat response. These effects were specific to the amygdala pathway as no difference in threat response was seen when CNO was infused into the NAc.

### Infusion of ketamine into the nucleus accumbens prevents the induction of anticipatory anhedonia

We have previously demonstrated that anticipatory anhedonia but not anxiety induced by activation of scACC-25 could be ameliorated by the fast-acting antidepressant ketamine, when given systemically to marmosets (8, 9). Given our present finding that anticipatory anhedonia could be reproduced by activation of the scACC-25-to-NAc pathway, we next determined whether the NAc and its associated neural network were a site of action for this selective effect of ketamine to ameliorate anticipatory anhedonia when the DREADD activator CNO was administered systemically. Our approach is supported by correlative human neuroimaging that implicates the ventral striatum in the ameliorative effect of ketamine on anhedonia (18) [note that rodent studies examining ketamine neurobiology have primarily focused on the medial prefrontal cortex and lateral habenula (19–21)].

**Fig 4:**
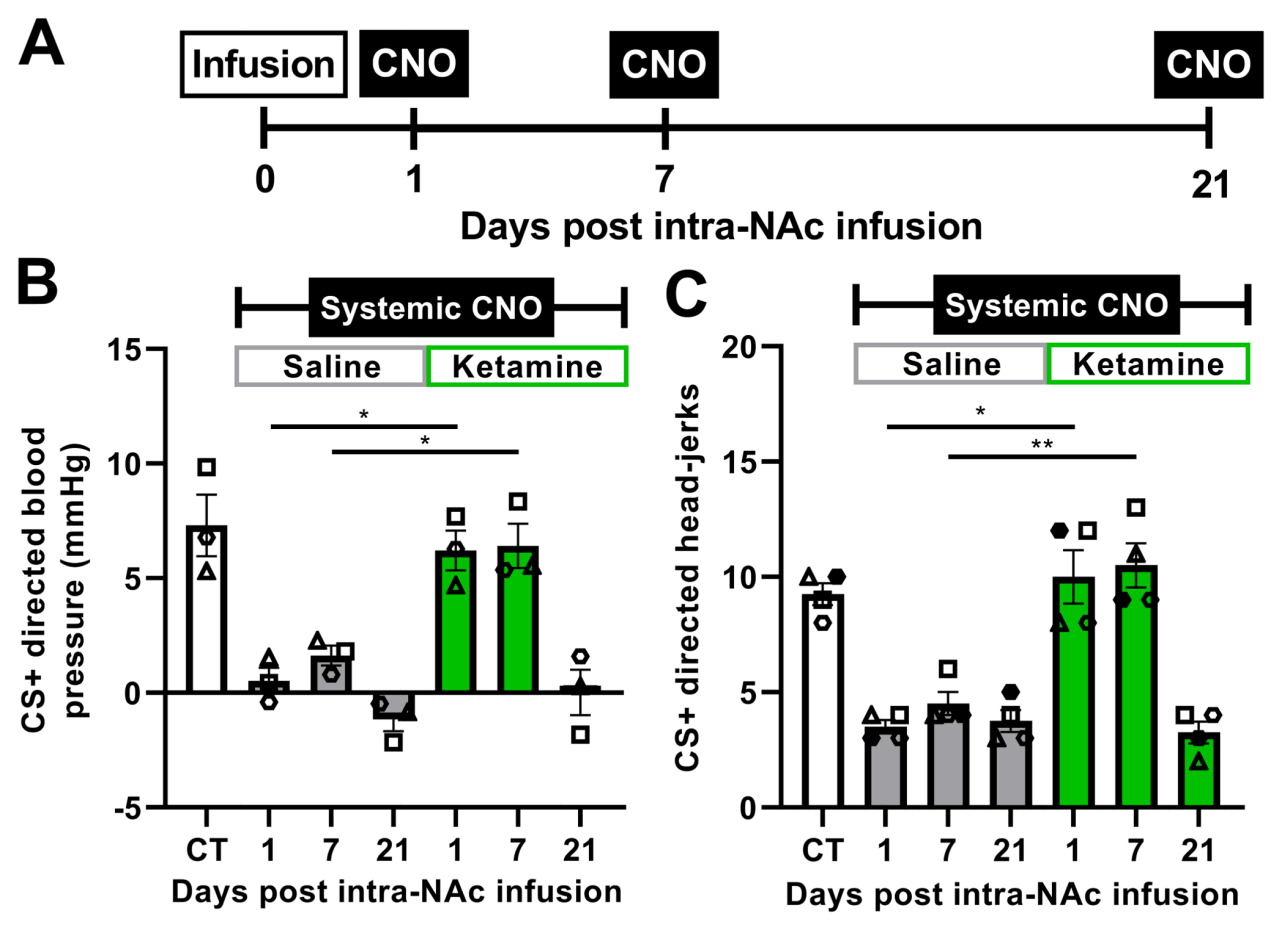
Prevention of anticipatory anhedonia by ketamine infusion into the marmoset NAc. (**A**) Ketamine (0.5μg, green) or saline as a vehicle control (grey) was infused into the NAc through intracerebral cannulae. CNO (10mg/kg, intramuscular) was administered peripherally 1, 7 and 21 days after the ketamine or saline infusion to blunt anticipatory arousal during appetitive Pavlovian testing. Shown are results for a reward-associated conditioned stimulus after CNO injection (CS+). (Data for CS- and unconditioned stimulus are presented in Fig. S10). (**B**) Prior to any treatment (CT), marmosets displayed directed blood pressure changes after a reward-associated conditioned stimulus (CS+). CNO blunted this anticipatory arousal at all timepoints following the saline infusion (grey bars). In contrast, ketamine (green bars) blocked the CNO-induced blunting on days 1 and 7 post-infusion, with this effect lost at day 21 post-infusion (n=3, two-way rmANOVA, treatment*time interaction, F_(2,4)_=8.248, p=0.038, η^2^=0.81; Sidak-corrected posthoc: 1day ketamine vs saline, p=0.047, *d*=4.5; 7 days ketamine vs saline, p=0.037, d=3.7). (**C**) Ketamine also blocked CNO-induced blunting of head-jerk behaviors at days 1 and 7 post-infusion but not day 21 post-infusion, whereas CNO-induced blunting of head-jerk behaviors occurred across all timepoints after saline infusion (n=4, two-way rmANOVA, treatment*time interaction, F_(2,6)_=17.156, p=0.003, η^2^=0.85; Sidak-corrected posthoc: 1day ketamine vs saline, p=0.012, *d*=3.9; 7 days ketamine vs saline, p=0.002, *d*=3.9). Mean data are displayed with SEM error bars and individual data points are shown. Significant Sidak-corrected comparisons indicated by p<0.05* and p<0.01**.

We infused either ketamine (0.5ug) or saline into the NAc of marmosets and measured reward anticipation in the appetitive Pavlovian task at 1, 7 and 21 days post-infusion (Fig S8), timepoints that are relevant to the prolonged effectiveness of ketamine in the clinic (Fig 4A, 22). At these timepoints, we administered CNO (10 mg/kg) systemically to activate all scACC-25 projections, including the projection to NAc, and induced anticipatory anhedonia comparable to that seen with pharmacological scACC-25 overactivation when saline (not ketamine) was infused into the NAc (Fig. 4B,C) (8). In a separate cohort of marmosets (Table S2, Supplementary data), we controlled for off-target systemic CNO effects by demonstrating that there was no effect on performance in either of the behavioral tests when CNO (3 or 10 mg/kg) was administered systemically prior to DREADD delivery to the brain (Fig S9).

Prior to any drug treatment or infusions, marmosets showed marked anticipatory cardiovascular (n=3, Fig 4B) and behavioral arousal (n=4, Fig 4C) to the reward-associated conditioned stimulus in a control session (CT, white bars). We then established the effects of systemic CNO administration on anticipatory arousal across the 3 time points (1, 7 and 21 days post infusion) by infusing saline into the NAc as a control. This allowed us to directly compare each saline and ketamine timepoint, but also controlled for the effects of the infusion process itself. Following the saline infusion, systemic CNO administration (10mg/kg, intramuscular) induced blunting of anticipatory cardiovascular and behavioral arousal at all 3 time points (Fig. 4B, C; grey bars). In contrast, the ketamine infusion prevented the blunting effect of CNO administered systemically on anticipatory cardiovascular and behavioral arousal at days 1 and 7 post-infusion, with marmosets displaying intact anticipatory blood pressure and head-jerk responses (green bars). These effects did not extend to 21 days post-infusion, with the return of CNO-induced blunting of reward anticipation at this timepoint. These effects on cardiovascular and behavioral anticipation occurred without any changes in response to the non-reward associated conditioned stimulus, the amount of reward consumed or the blood pressure response to consuming the reward (Fig S10).

## Discussion

Anhedonia and enhanced negative emotion commonly co-occur in patients with MDD. Here, using chemogenetics, we demonstrate that overactivation of non-human primate scACC-25 induced specific components of both anhedonia and anxiety through separate pathways connecting scACC-25 to the NAc and amygdala, respectively. The finding that anticipatory but not consummatory appetitive responses were blunted after overactivation of the scACC-25 in marmosets provided additional validity to these findings because deficits in anticipatory and motivational aspects of reward are more readily observed in patients with MDD than deficits in the consummatory ‘liking of reward’ (3, 15, 23).

Given that the amygdala and NAc are both implicated in appetitive behavior (24, 25), overactivation of neural projections to either structure could be predicted to blunt anticipatory arousal, which, however, did not occur. Instead, scACC-25 projections to the NAc but not the amygdala, were selectively responsible for blunting appetitive, anticipatory cardiovascular and behavioral arousal in marmosets, presumably by inhibiting expression of these conditioned responses. Given that the NAc is a critical hub mediating anticipatory and motivational aspects of reward-related behavior and is almost exclusively GABAergic (26, 27), the scACC-25 excitatory afferents may be selectively impacting inhibitory function within this ventral striatal network that results in blunting of appetitive behavioral and cardiovascular responses. This is consistent with the reported hypoactivation of NAc in patients with MDD in response to a reward (13) and the negative correlation between anhedonia severity and ventral striatal activity (28). However, the considerable complexity within the NAc inhibitory microcircuitry is still to be fully elucidated.

Relevant to the understanding of ketamine’s efficacy in patients with MDD, ketamine infusion into the NAc protected marmosets from the anhedonic-like effects of scACC-25 overactivation for more than one week. This parallels results reported previously in humans, and more recently in marmosets, following systemic ketamine administration (8, 22, 29, 30). Identification of the neural circuit mechanism through which ketamine acts to mediate its anti-anhedonic effects is critical for developing more effective long-term treatments for patients with MDD. Here we highlight the NAc as one potential site of action for ketamine. Either plasticity changes within the NAc overall or within the scACC-25 to NAc pathway may have mediated these longer-term ketamine effects by lessening the deleterious impact of scACC-25 overactivation. Rodent studies highlight regional differences in plasticity, with ketamine increasing plasticity in the mPFC (31), whilst reducing plasticity in the NAc (32). If these effects translate to nonhuman primates, ketamine may be blocking the induction of anticipatory anhedonia through reducing overall NAc responsivity to scACC-25 activation or by directly limiting the excitatory drive produced from the neurons connecting the scACC-25 to NAc. The translational potential of these findings are suggested by the reduction in scACC-25 hyperactivity to positive, but not negative, incentives in patients with MDD after acute ketamine administration (30).

Neural projections from the scACC-25 to the NAc were not, however, responsible for the heightening of reactivity to an uncertain threat by scACC-25 overactivation in our study (9), despite the NAc being associated with cue-induced threat responses (11). Instead, our study revealed that neural projections from the scACC-25 to the amygdala mediated this effect, the amygdala being important for identifying the emotional significance of stimuli in the environment (10). Our findings are supported by our previous ^18^F-flurodeoxyglucose PET imaging study in marmosets demonstrating increased activity in the amygdala following pharmacological scACC-25 overactivation in a threatening context (9). Tracing studies in macaques have shown that area 25 has one of the most dense reciprocal projections with the amygdala of all frontal lobe regions (33) and innervates the basomedial and basolateral nuclei (as evidenced here in marmosets) to enhance amygdala output. This contrasts with the purported analogous rodent structure, the infralimbic cortex, which has excitatory projections that innervate the inhibitory intercalated masses and act to reduce emotional output via their projections to the medial part of the central amygdala nucleus (34). These species differences in amygdala connectivity further illustrate the critical importance of studying these processes in non-human primates when translating cortical function to humans, as the cortical circuitry of nonhuman primates is more similar to humans than that of rodents (1, 2).

There have been few studies using DREADDs in non-human primates thus far, and these have been primarily limited to proof of principle studies or studies investigating sensory processing or cognitive decision making (35–37). In the current study, whilst aiming to recapitulate core aspects of affective disorders (38) with the prototypic activator CNO, we also used a new DREADD activator, deschloroclozapine, that enabled high efficacy at low doses (39), further supporting our dissection of the specific pathways mediating distinct symptom domains.

There are several limitations to our study. First, the number of marmosets used within these studies is relatively small, therefore care must be taken not to over interpret the results. It is worth noting, however, that these findings are building on previously published results (8, 9) and replication of those prior results was a necessary first step. Second, potential contributions from other scACC-25 neural pathways through back propagation of the signals in the amygdala and NAc cannot be ruled out. However, back propagation after indirect increases in excitability at the nerve terminals caused by CNO via G-protein coupled receptors, seems less likely. Third, the physiological effects of terminal activation by the DREADD hM3Dq have not been fully established, especially in non-human primates. Future studies will need to characterize how the hM3Dq DREADD impacts excitability within nerve terminals. Finally, our study should not be interpreted as suggesting that the scACC-25-to-amygdala or scACC-25-to-NAc neural pathways are specific, respectively, for threatening and rewarding stimuli in general. The differential impact of these neuronal pathways from scACC-25 on reward and threat responses may be a consequence of the differential cognitive processes engaged in response to the unambiguous cues versus ambiguous cues used here in the appetitive Pavlovian reward test and human intruder test, respectively. Future studies should control for cue ambiguity across rewarding and threatening contexts to disambiguate these two variables.

To conclude, we propose that the direct causal functions of this scACC-25 neural network need to be examined through intervention studies in non-human primates as the structural organization of the nonhuman primate vmPFC is more similar to that of human than the corresponding region in the rodent brain (1, 40). Previous work has established a distinct causal contribution of scACC-25, area 32 and area 14 within the vmPFC to reward-associated and threat-associated behaviors in the marmoset (2, 9, 41), however, detailed analysis of specific pathways has been lacking. The experimental approach adopted here is especially important given the variability in the direction of dysfunctional activity reported within the vmPFC or scACC in functional neuroimaging studies of patients with psychiatric disorders. Our work has laid the foundation for future DREADD experiments to further reveal the pathway-specific dysregulation in nonhuman primates that underlies specific components of neuropsychiatric symptoms.

## Materials and Methods

### Study Design

The objective of this study was to examine how activation of distinct scACC-25 neuronal pathways in the marmoset brain directly contribute to components of anhedonia and anxiety, and whether the pathway identified as inducing an anhedonic-like response contributes to the ability of the fast-acting antidepressant ketamine to treat anhedonia. The main study to address this core objective used five marmosets. A further six marmosets were used to establish the specificity of CNO to induce anhedonia and anxiety through its activation of the DREADD hM3Dq in scACC-25, when administered systemically (Fig S9). All marmosets were allocated to these studies based on them displaying average levels of trait anxiety determined by their response in a human intruder test carried out in early adulthood (~22-24 months). All behavioral measures throughout these studies were analyzed by an individual blinded to the treatment. Apart from one marmoset’s cardiovascular data, that could not be collected due to a telemetry communication failure, no outliers were excluded from any dataset. All animals acted as their own controls using a within subject design. All surgical procedures were performed under aseptic conditions and marmosets received 7-10 days recovery before any further procedures. All procedures were carried out in accordance with the UK Animals (Scientific Procedures) Act 1968 and the University of Cambridge Animal Welfare and Ethical Review Board.

### Animals

Eleven experimentally naïve common marmosets (Callithrix jacchus; five across the main study, six within the systemic CNO control study in Fig S9; see Table S2) bred on site at the University of Cambridge Marmoset Breeding Colony were housed in male-female pairs (males were vasectomised). The room maintained a 12 hour light-dark schedule (7am lights on; 7pm lights off; 30-minute phased change). Marmosets had ad libitum access to water and were fed a varied diet of MP.E1 primate diet (Special Diet Services, UK), carrots, fruit, rusk, malt loaf, eggs and bread. Their cages (2.80 x 1.20 x 0.98m) contained a nest-box with environmental enrichment.

The five marmosets in the main study consisted of two females and three males and the six marmosets used to generate data in Fig S9 consisted of four females and two males (see Table S2). All marmosets received a cardiovascular telemetric probe implanted into their descending aorta and were trained on an appetitive Pavlovian discrimination task to assess reward anticipation and were tested on an uncertain threat test, known as the human intruder test. For the main study, marmosets 1-5, received bilateral infusion of a DREADD-containing virus targeting scACC-25 (AAV8-CaMKIIa-HA-hM3Dq, VectorBuilder). Following recovery, they then received bilateral cannulation of the nucleus accumbens core and basal amygdala. For control experiments presented in Fig S9, marmosets initially received systemic administration of the DREADD activator clozapine N-oxide to assess off-target effects on the Pavlovian discrimination and human intruder behavioral tests. Marmosets 7-11 received a bilateral infusion of a DREADD-containing virus targeting scACC-25 (AAV8-CaMKIIa-HA-hM3Dq-IRES-mCitrine, UNC Vector Core) enabling analysis of the systemic effects of DREADD-mediated activation of scACC-25 on the appetitive Pavlovian discrimination and human intruder tests.

### Surgery to insert a telemetry probe

One day before surgery, marmosets received the antibiotic enrofloxacin (Bayer, 0.5mg, p.o.). Immediately prior to surgery, marmosets were premedicated with ketamine hydrochloride (Ketavet, 10mg, i.m.) before receiving the analgesic meloxicam (Boehringer Ingelheim, 0.075 mL of 2mg/mL solution, s.c.) and enrofloxacin (Bayer, 5mg, s.c.). They were then intubated and maintained on 2-2.5% isoflurane in 0.3-0.4l/min O_2_. They were monitored throughout surgery using pulse-oximetry, capnography (Microcap Handheld Capnograph) and a rectal temperature probe (TES-1319 K-type digital thermometer). The aorta was localized within the marmosets’ abdomen and the end of the HD-S10 telemetry probe (Data Sciences International) was inserted into the descending aorta just above the aortic bifurcation, with blood occluded for no more than 3 minutes. The probe was then sutured in place within the abdomen. Following recovery, marmosets received the analgesic meloxicam (Boehringer Ingelheim, 0.15mg, p.o.) for 3 days post operatively.

### Bilateral infusion of AAV-containing hM3Dq DREADD

Following sedation and intubation as described above, marmosets were placed in a stereotaxic frame adapted for marmosets (David Kopf Instruments). Through small holes drilled in the skull, the dura was finely pierced by a sterile needle before a sterile 26-gauge injector with a 30° bevel (Plastics One) was lowered into scACC-25 (2 AP co-ordinates, anteroposterior [AP], +14.0 mm and +13.5mm; lateromedial [LM], ±0.7 mm). Coordinates were adjusted in situ based on cortical depth within the prefrontal cortex at +17.5 AP, −1.5 LM as previously reported (42), with a second adjustment within the vmPFC if required to ensure a depth between 8.9 and 9.3 mm at +14.0 AP, −1.0 LM (41). The injector was connected to a gas tight 10ul Hamilton syringe, with infusions of the AAV containing the hM3Dq DREADD at 0.1ul/min (2.5 x 10^9^ viral genomes/ul, 1ul each side/AP co-ordinate). After infusions, the injector was left in place for 10 minutes to allow for diffusion before being slowly removed. After all infusions, skull holes were filled with a small portion of tissue adhesive (Vetbond) and the skin was sutured. Postoperatively, marmosets received meloxicam as indicated above.

### Surgery to insert a cannula

Marmosets were anesthetized and intubated as described above and were placed in the marmoset stereotaxic frame. Cannulae (Bilaney Consultants, UK) were implanted targeting the nucleus accumbens core (26-gauge single cannulae, 6mm protrusion from the guide base, AP +12.3, LM±2.2) and central amygdala (26-gauge single cannulae, 15mm protrusion from the guide base, AP +9.1, LM ±5.6) taking into account any previous in situ co-ordinate adjustments for each marmoset. Postoperatively, marmosets received meloxicam as indicated above. Weekly, the cannulae and mount were cleaned so the site remained infection free, with caps and dummies replaced to maintain patency.

### Drug administration

Prior to any drug treatment, marmosets were habituated to the handling procedure for either central or peripheral drug administration, with marmosets held gently by an assistant familiar to them.

For central infusions, the caps and dummies were removed from the guide and the site was cleaned with 70% isopropyl alcohol. Sterile injectors (Bilaney Consultants, UK) connected to a 10ul gas tight syringe pump was inserted into the guide. The length of the injector was determined by the cortical depth and the placement of the guide cannula during surgery. Bilateral infusions were carried out for 2 minutes at a rate of 0.5ul/min, with the injector left in place for an additional minute to allow drug diffusion before removal. Sterile dummies and caps were then replaced, and the marmoset was returned to their homecage for the appropriate pre-treatment period. CNO (Tocris, UK) was infused at 3μM (43), whilst DCZ (HelloBio, UK) was infused at 100nM (44). Central CNO and DCZ were initially dissolved in dimethyl sulfoxide (DMSO, Sigma-Aldrich) before being diluted to their appropriate concentration with final solutions being 1% DMSO saline, with a pretreatment time of 30 minutes. Racemic ketamine (Tocris, UK) was dissolved in sterile saline and infused at 0.5ug/ul (similar to 21). CYP3A4, the main cytochrome P450 enzyme ortholog for ketamine (45), is minimally expressed within the marmoset brain (46) therefore effects of ketamine metabolites are expected to be minimal. Drug infusion order were counterbalanced across subjects.

For systemic treatment, CNO (3 or 10mg/kg) was dissolved in 5% DMSO saline for 0.1ml intramuscular injections. CNO has been used previously in non-human primates with a pre-treatment time of 1hr (47), with validation of DREADD-specific effects provided in Fig S9.

### Behavioral testing

Prior to any behavioral testing, marmosets were trained to enter a transparent Perspex travel box (240 × 230 × 200mm) in which they were transported to the behavioral testing chamber which was used for the appetitive Pavlovian discrimination test. The human intruder test was conducted within the home cage. Across all behavioral paradigms, within subject controls were used throughout.

#### Appetitive Pavlovian Discrimination test

This test has an extensively detailed protocol published previously (48). Marmosets were trained in a bespoke testing chamber to associate two auditory cues, acting as Pavlovian conditioned stimuli (CS, 20s in length) associated with distinct unconditioned stimuli (US); the CS− predicted a door opening to reveal a transparent window through which could be seen an empty food box on one side (US-) and the CS+ predicted the door opening on the opposite side, providing access to marshmallow (US+; ∼6-8 g). These CSs continued throughout their associated US period (120s). Daily testing sessions (5 days a week) included 1-2 CS trials; either single CS- or CS+, double CS-, or a CS- followed by CS+. Inter-CS intervals were 70-110s. Marmosets received five CS+ trials over 2 weeks.

Successful discrimination was defined as increased CS+ directed (CS+ minus 20s baseline period) mean arterial pressure (MAP) and rapid head-jerk responses relative to the CS− directed response (CS− minus 20s baseline period) - scored by an experimenter blind to treatment. Drug treatments occurred prior to two-trial sessions containing a CS− trial followed by a CS+ trial. Treatments occurred at least 1 week apart, with non-drug test sessions occurring in between to confirm the return of normal discrimination. Consumption measurements were also calculated including a MAP response to the US+ compared to the CS+ (US directed) and reward consumption (grams of marshmallow eaten).

As a control, during the CS periods locomotor activity was scored by recording the amount of time marmosets moved locations within the apparatus, similar to that conducted for the human intruder test (49). Specifically, this is the total time in which all 4 limbs were in motion during the CS periods. To compare locomotor activity and cardiovascular arousal during the CS periods we have plotted CS-directed MAP responses with this activity in Fig S4.

#### Human Intruder test

Testing was conducted similarly to that previously described (49). Marmosets were separated from their cage mate into the upper-right quadrant of their home cage 8 minutes before the end of the drug pre-treatment time. Following this, an unfamiliar human intruder entered the room and stood 40cm from the cage, maintaining eye contact throughout the 2-minute test period. The intruder was a researcher wearing a realistic human mask (Masks Direct) unfamiliar to the marmoset and wearing familiar scrubs. The order of masks and infusions were counterbalanced across marmosets with at least 2 weeks between each test. Behavior was recorded using a camera (GoPro Hero 5) and a microphone was used to record vocalisations (Sennheiser MKE 400).

#### Histological analysis

Marmosets were premedicated with ketamine hydrochloride (10mg, i.m.) and subsequently euthanized with sodium pentobarbital (Dolethal, 200mg i.v.). Marmosets were then transcardially perfused with ice cold 0.1M phosphate buffer saline (PBS; Sigma-Aldrich) followed by 4% formaldehyde solution (VWR international). The brain was removed, placed in 4% formaldehyde solution overnight, then 0.01M PBS-azide for 48 hours and finally 30% sucrose for 72 hours. The brain was sectioned using a freezing microtome (40μm) in 5 series. One series were used to assess HA-tag staining whilst another was stained with Cresyl violet (FD Cresyl Violet, #PS102-01, FD Neurotechnologies). Standard brightfield microscopy was used for cannulae and infusion localization.

For HA-tag imaging, sections were washed in 0.01M PBS-Triton X100 (PBS-T; Sigma-Aldrich, 0.3% v/v, 3 x 10mins) and then blocked for two hours with 3% normal goat serum/1% bovine serum albumin solution (w/v in 0.01M PBS-T) before being incubated overnight with primary antibody (1:400 anti HA-tag primary antibody, Cell Signaling Technology Cat# 3724, RRID:AB_1549585). The following day, sections were washed in PBS-T and then incubated for 2 hours with the secondary antibody (1:1000 goat anti-rabbit Alexa 488, Abcam Cat# ab150077, RRID:AB_2630356) after which sections were washed in 0.01M PBS, mounted onto Superfrost slides using Fluorosave mountant and coverslipped. Sections were visualized using a M205FA stereo microscope (Leica, UK), DFC7000T camera (Leica, UK) and LASX software (version 2.004). HA-tag immunofluorescence images were taken with an X-Cite 200DC illuminator (Lumen Dynamics, UK) with the GFP1 filter (Leica, UK). Expression of DREADD fused HA-tag fluorescing cell bodies were localized within scACC-25 alone for all subjects, with the anterior and posterior infusions enabling expression to span the rostrocaudal extent of scACC-25 with expression contained within sections +12.8 AP to +14.2 AP of the marmoset atlas (50).

### Data and statistical analysis

All figures were produced within GraphPad Prism 9, with statistical analysis within IBM SPSS statistic (v27, IBM, USA). Significance was set at *α*=0.05 in all cases. For all analysis of variance (ANOVAs), F, p and partial eta squared (η^2^) values are reported throughout for main effects and interactions where appropriate with the Huynh-Feldt correction used when sphericity was violated. Appropriate post-hoc comparisons used Sidak-correction with p value and Cohen’s *d* reported where appropriate. Statistical significance is indicated by * for p<0.05, ** for p<0.01 and *** for p<0.001 in all figures.

#### Appetitive Pavlovian Discrimination test

Data analysis related to this test has been extensively described in an experimental protocol (48). Cardiovascular data were continuously transmitted by the telemetric probe to the receiver during testing for offline analysis using Spike 2 (Version 11, Cambridge Electronic Design). Data were reliable overall with minimal data loss throughout experimentation, except for a single marmoset whose telemetric data showed abnormal values throughout due to communication failure with the receiver and so these data were not included in any cardiovascular dataset. Mean arterial pressure (MAP) was calculated from the systolic and diastolic blood pressure events in each cardiac cycle using the formula MAP = dBP + 1/3(sBP – dBP). MAP was used as the principal cardiovascular measure as it was more consistent during conditioning than, for example, heart rate, consistent with previous reports following vmPFC or scACC-25 manipulation (8, 41).

For the scACC-25 pathway activation study, conditioned stimulus-directed cardiovascular responses and head-jerk behaviors were analysed using within subject factors of treatment (vehicle and CNO), region (NAc and amygdala) and conditioned stimulus (CS- and CS+). For the ketamine study, each conditioned stimulus type was analysed separately by repeated measures ANOVA, using within subject factors of treatment (ketamine and saline) and time (1, 7 and 21 days post-infusion). For unconditioned stimulus data across these experiments, repeated measures ANOVA used all non-conditioned stimulus factors from each experiment. Correlations between locomotor activity during the conditioned stimulus periods and conditioned stimulus-directed cardiovascular responses were calculated using Pearson’s correlation (Fig S4). Degrees of freedom, r and p values are reported.

Data showing the validation of systemic CNO treatment effects used separate repeated measures (rm)ANOVA for the pre-DREADD and post-DREADD phases. Conditioned stimulus-directed data (head-jerk behaviors and cardiovascular responses) were analyzed using within subject factors of treatment (vehicle, 3mg/kg CNO and 10mg/kg CNO) and conditioned stimuli (CS- and CS+). For unconditioned stimulus data (reward consumption and cardiovascular responses), a within subject factor of treatment was used (vehicle, 3mg/kg CNO and 10mg/kg CNO).

#### Human intruder test

Individual behaviors during the human intruder test were scored offline by an individual blinded to treatment using Jwatcher software (UCLA and Macquarie University). Specific behaviors scored included the position of the marmosets within the cage, head bobs and body bobs, jumps and locomotion. Vocalisations, including tsik, egg, tsik-egg and tse-egg calls were scored using Audacity. A composite threat response score was derived from these measures based upon an exploratory factor analysis of the responses of 170 marmosets on this test. This analysis identified a single factor that accounts for 39.7% of the total variance and has been described extensively (49). The threat response score and individual measures were analysed for normality using the Shapiro-Wilk test, with non-normal data analysed using non-parametric methods described as follows.

The threat response score and individual behaviors from the scACC-25 pathway analysis were analysed using a repeated measures ANOVA with treatment (vehicle and CNO) and region (NAc and amygdala) as within subject factors. Non-parametric data were analysed using the Friedman test across all treatment and region groups, with chi-squared and p-values reported. A summary of the individual measures and statistics are provided in Table S1.

In the systemic CNO validation data (Fig S9), the threat response score and percentage time spent at the front of the cage were analyzed using a linear mixed effects model using Satterthwaite approximations with study phase (pre-DREADD and post-DREADD) and treatment (vehicle and CNO) as factors. Due to marked individual differences in the reactivity of marmosets to the unknown human in this study (threat response range, pre-DREADD: -1.7 to 0.52, post-DREADD: -1.2 to -0.5), a difference score between the CNO and vehicle data was derived (score calculated as CNO – vehicle). These difference scores were analysed using a one sampled t-test versus a hypothetical mean of 0, with a comparison between phases using an unpaired t-test with Welch’s correction.

## Supplementary Material

Supplementary materials

## Data Availability

All data associated with this study are in the paper or supplementary materials. Source data are provided in Data File S1 and in a Mendeley Data repository (https://doi.org/10.17632/nm48c2y3gx.1). AAVs used in this paper were obtained from VectorBuilder and the UNC Vector Core. The VectorBuilder plasmid sequence can be found at: https://en.vectorbuilder.com/vector/VB191022-1031hqp.html. Plasmids for the other AAV vector can be acquired through Addgene (http://www.addgene.org/50466/).

## Editor’s summary

### Dissecting the neural circuitry of depression

There is still much to learn about the brain circuits involved in depression and anxiety. Here, Wood and colleagues demonstrate that specific connections of a brain region called area 25 directly affect anxiety and anhedonia in marmosets. Using a chemogenetics strategy, the authors showed that activation of area 25 connections to the nucleus accumbens resulted in anhedonia, whereas activation of area 25 connections to the amygdala induced anxiety in the animals. Moreover, the nucleus accumbens was a target for the response of marmosets to the fast-acting antidepressant ketamine. These findings may help to elucidate more effective treatment strategies for depression and anxiety.

## One-Sentence Summary

Separate area 25 brain circuits drive reward and threat-associated responses in nonhuman primates.
