# Supplementary material for: Chemogenetics identifies separate area 25 brain circuits involved in anhedonia and anxiety in marmosets
Source: Sci Transl Med. Author manuscript; Available in PMC 2023 Apr 24. (PMC7614473; doi:10.1126/scitranslmed.ade1779)
Supplement: Supplementary materials [file EMS173660-supplement-Supplementary_materials.docx]

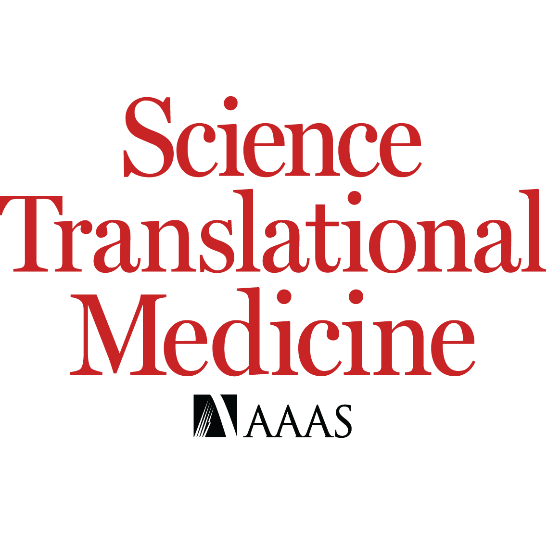


Supplementary Materials for

Chemogenetics identifies separate area 25 brain circuits involved in anhedonia and anxiety in marmosets

Christian M. Wood^1,2*^, Laith Alexander^1,2^, Johan Alsiö^2,3^, Andrea M. Santangelo^1,2†^, Lauren McIver^1,2^, Gemma J. Cockcroft^1,2^, Angela C. Roberts^1,2,4*^.

Correspondence to: [cmw84@cam.ac.uk](mailto:cmw84@cam.ac.uk); [acr4@cam.ac.uk](mailto:acr4@cam.ac.uk)

**This PDF file includes:**

Figs. S1 to S10

Tables S1 to S2

Supplementary Figures


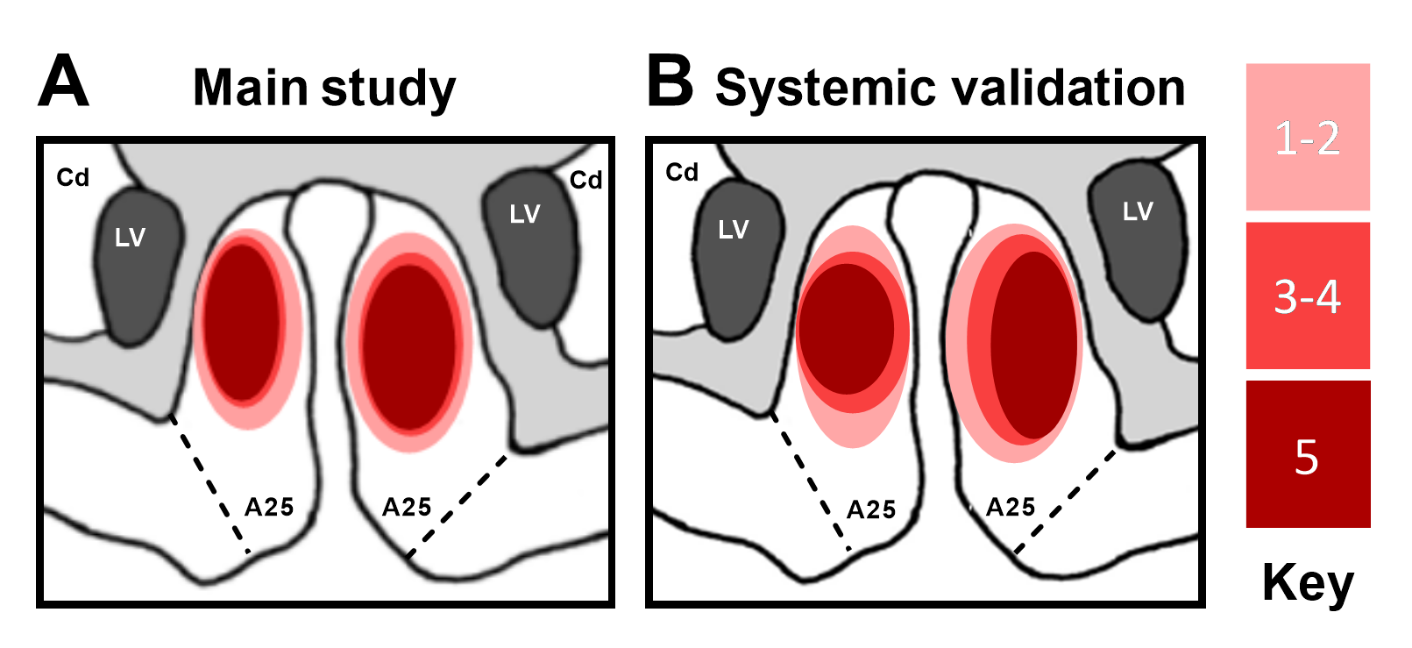


Fig S1. Schematic summary of DREADD virus infusion sites within scACC-25. (A) Summary of main study subjects 1-5 illustrating post-mortem DREADD fused HA tag visualization for each individual marmoset overlaid on an Area 25 schematic, with brain regions labelled based on the marmoset atlas (*50*; AP+13.8; A25, scACC-25; Cd, caudate; LV, lateral ventricle). Deeper red colour indicates overlap in expression localization in 5 marmosets with lighter shading indicating overlap in fewer (3-4 and 1-2) marmosets, respectively, (see key on the right). (B) Similar summary schematic for the DREADD expression in the systemic validation subjects utilizing HA tag immunofluorescence.


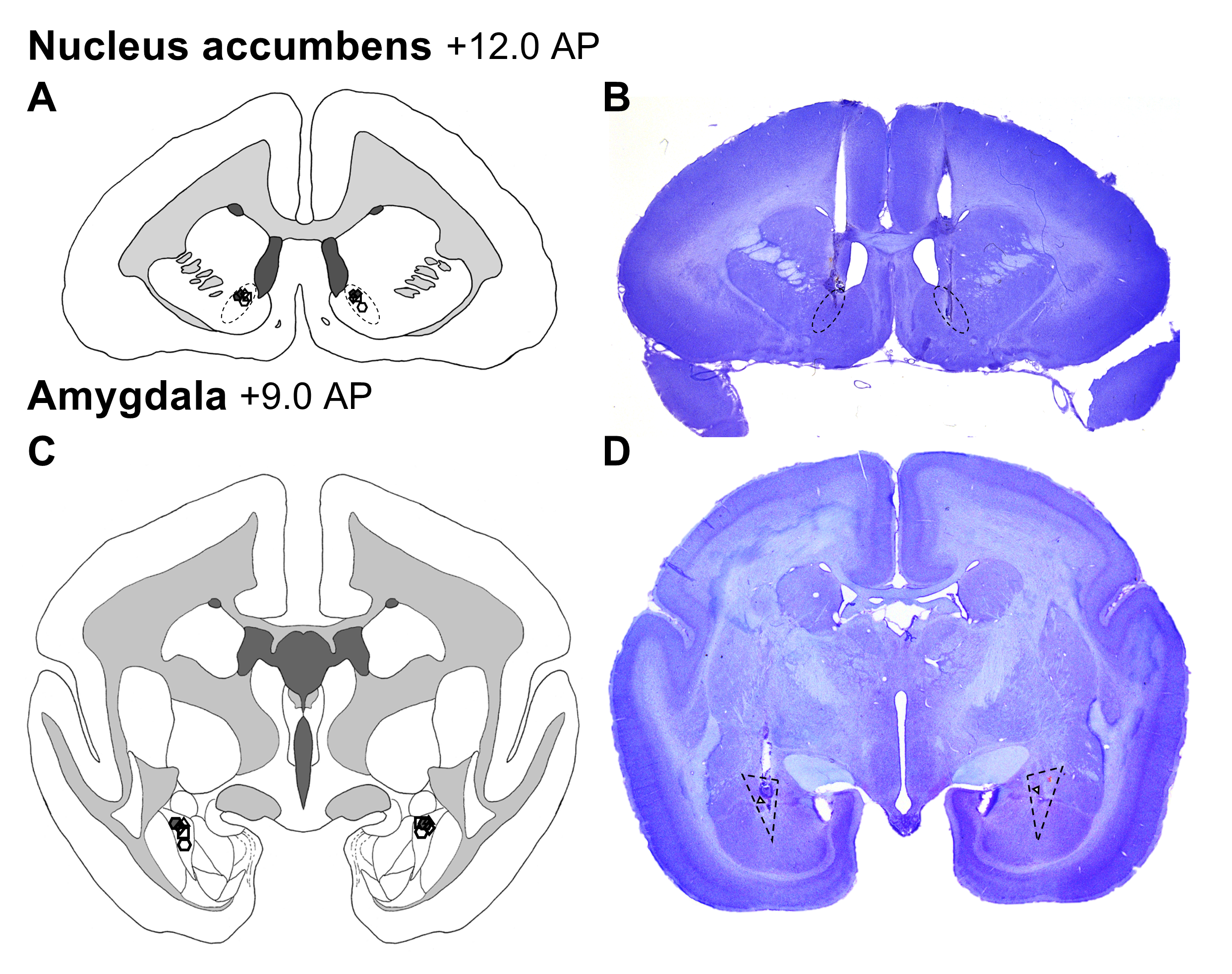


**Fig S2. Post-mortem infusion sites within the nucleus accumbens and amygdala.** (**A**) Schematic indicating each subject’s infusion site within the nucleus accumbens core (dashed oval, n=5). (**B**) Example cresyl stained section from subject 7 (hexagon) showing infusion site within the nucleus accumbens. (**C**) Schematic indicating each subject’s infusion site within the amygdala, with all sites located within the lateral sector of the basal nucleus of the amygdala. (**D**) Example cresyl stained section from subject 7 (hexagon) showing the infusions site within the amygdala with white arrows indicating the exact site.


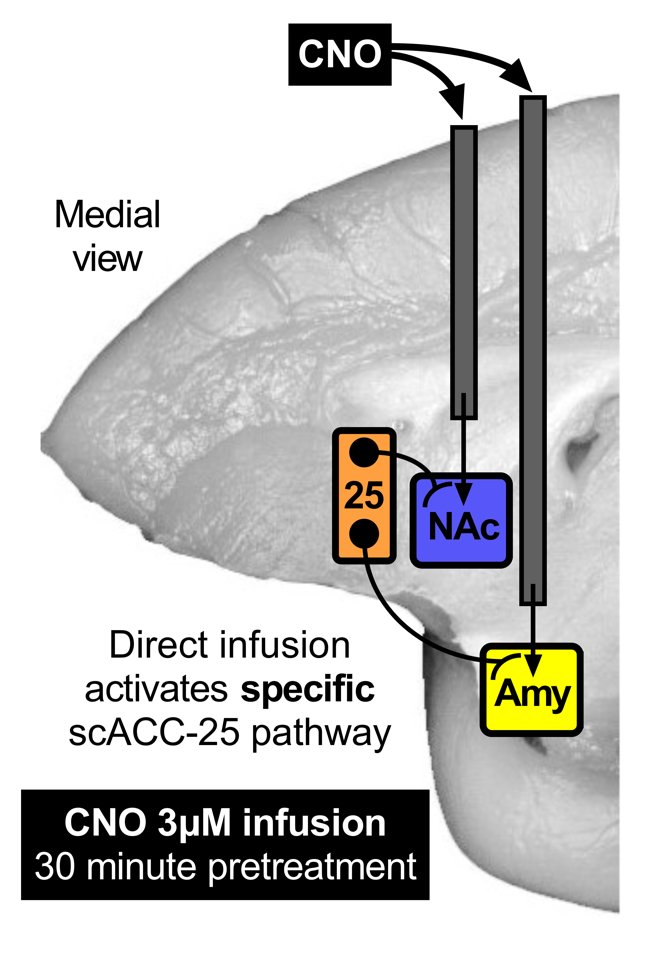


Fig S3. Schematic of scACC-25 pathway activation by CNO-mediated intracerebral infusion. With DREADDs expressed in output neurons of scACC-25, cannulae targeted the nucleus accumbens (NAc) core and basal amygdala (Amy) enabling activation of specific scACC-25 projections onto synaptic terminals through CNO infusion (3uM).


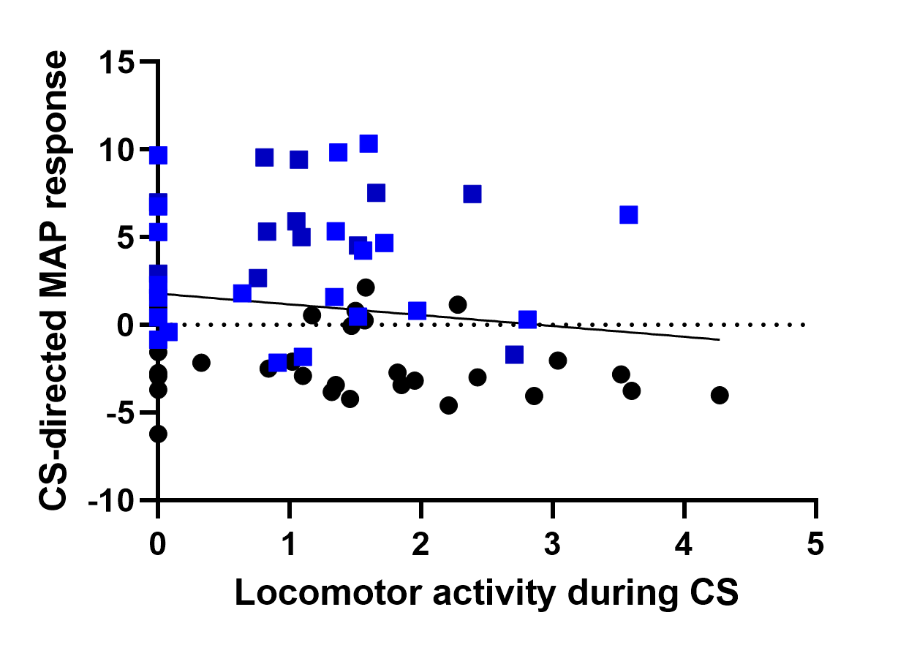


**Fig S4. Cardiovascular arousal to conditioned stimuli are independent of locomotor activity.** CS+ (blue square) and CS- (black circle) directed mean arterial blood pressure (MAP) responses from Fig 2 and Fig 4 are plotted against locomotor activity (LMA) during the CS periods. Correlation analysis between CS-directed MAP response and LMA highlighted no significant correlation between LMA and both CS types combined (black line, r_(64)_=-0.16, p=0.21), as well as each CS type individually (CS-, r_(31)_=-0.28, p=0.12; CS+, r_(31)_=0.031, p=0.86) suggesting the cardiovascular arousal to the CSs are independent of locomotor activity.

**
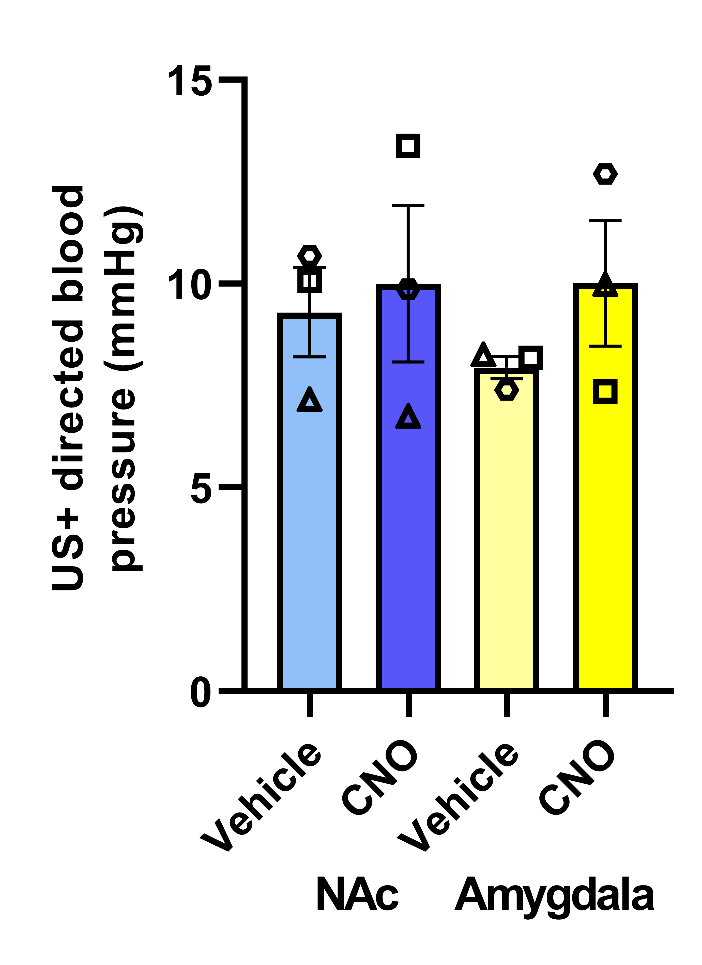
**

**Fig S5. Manipulation of scACC-25 pathways did not influence consummatory MAP responses.** DMSO (1%) saline vehicle or CNO (3uM) were infused into the nucleus accumbens or amygdala 30 minutes prior to testing on the appetitive Pavlovian conditioning paradigm. Activation of either pathway did not influence US+ directed MAP response (n=3, two-way rmANOVA, treatment*region, F<1, p=0.691). Data are displayed as means with error bars indicating SEM and individual data points for each subject.

**
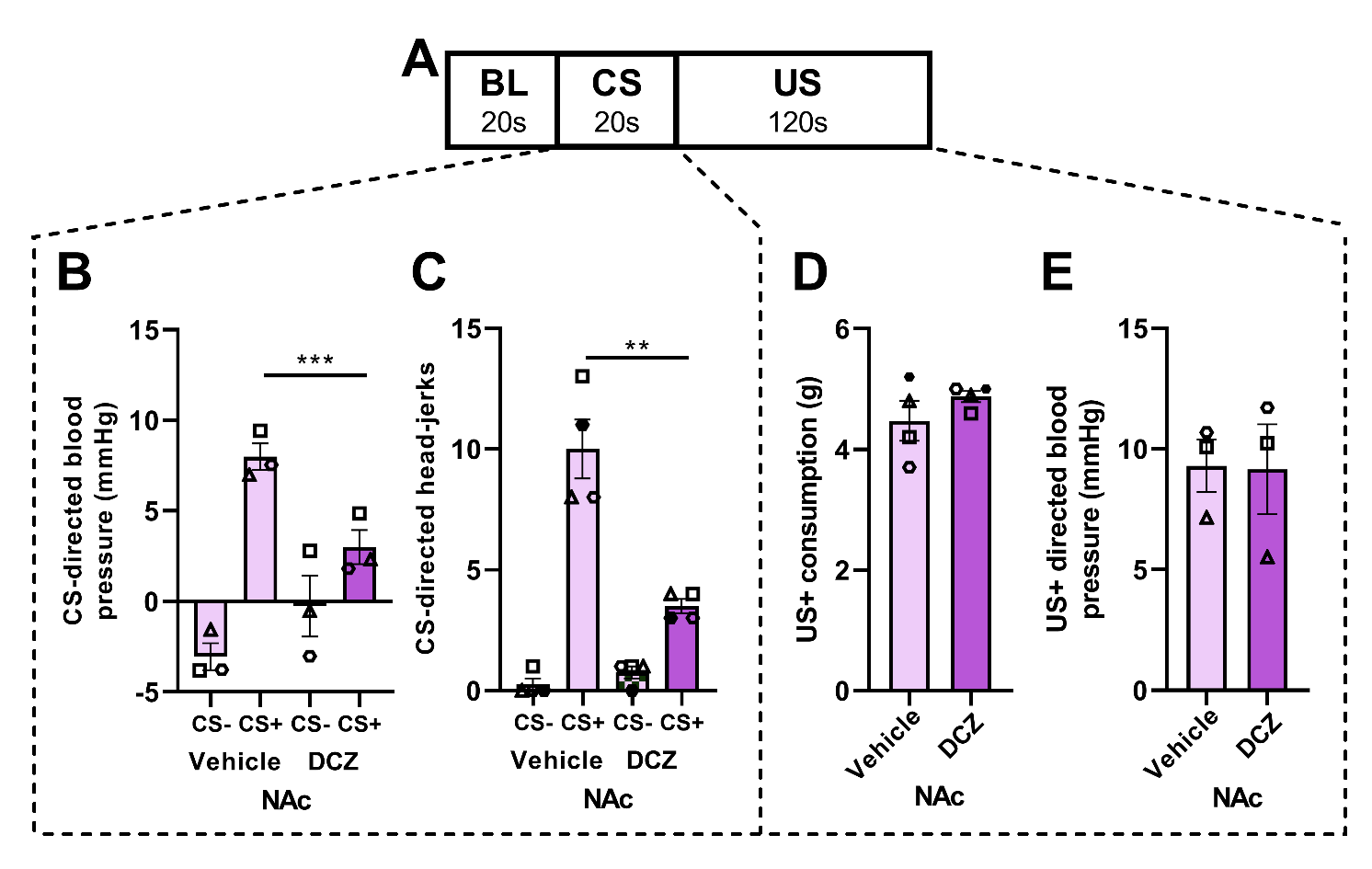
**

**Fig S6. Direct infusion of the novel DREADD activator deschloroclozapine (DCZ; 100nM) into the nucleus accumbens (NAc) blunted anticipatory cardiovascular and behavioural arousal whilst leaving consummatory responses intact.** (**A**) Trial schematic indicating length of each component with data from the CS and US periods indicated. (**B**) Direct infusion of DCZ (100nM) into the nucleus accumbens blunted CS+ directed MAP responses compared to vehicle with no effect on the CS- trial (n=3, two-way rmANOVA, treatment*CS, F_(1,2)_=24.523, p=0.038, η^2^=0.93; Sidak-corrected posthoc: CS+ Vehicle vs DCZ, p=<0.001, *d*=2.8). (**C**) CS+ directed head-jerks were also blunted by intra-accumbens DCZ infusion when compared to vehicle with no effect on the CS- (n=4, two-way rmANOVA, treatment*CS, F_(1,3)_=72.086, p=0.003, η^2^=0.96; Sidak-corrected posthoc: CS+ Vehicle vs DCZ, p=0.009, *d*=4.2). (**D**) Consumption of the marshmallow food reward (US+) was unaffected by the DCZ infusion (n=4, paired t-test, p=0.305). (**E**) Equally, the US+ MAP response was also unaffected (n=3, paired t-test, p=0.871). Data are displayed as means with SEM error bars and individual data points for each marmoset (subjects 1, 2, 3 and 5). Note that the cardiovascular probe of subject 3 stopped communicating with the receiver so only behavioural responses are shown.


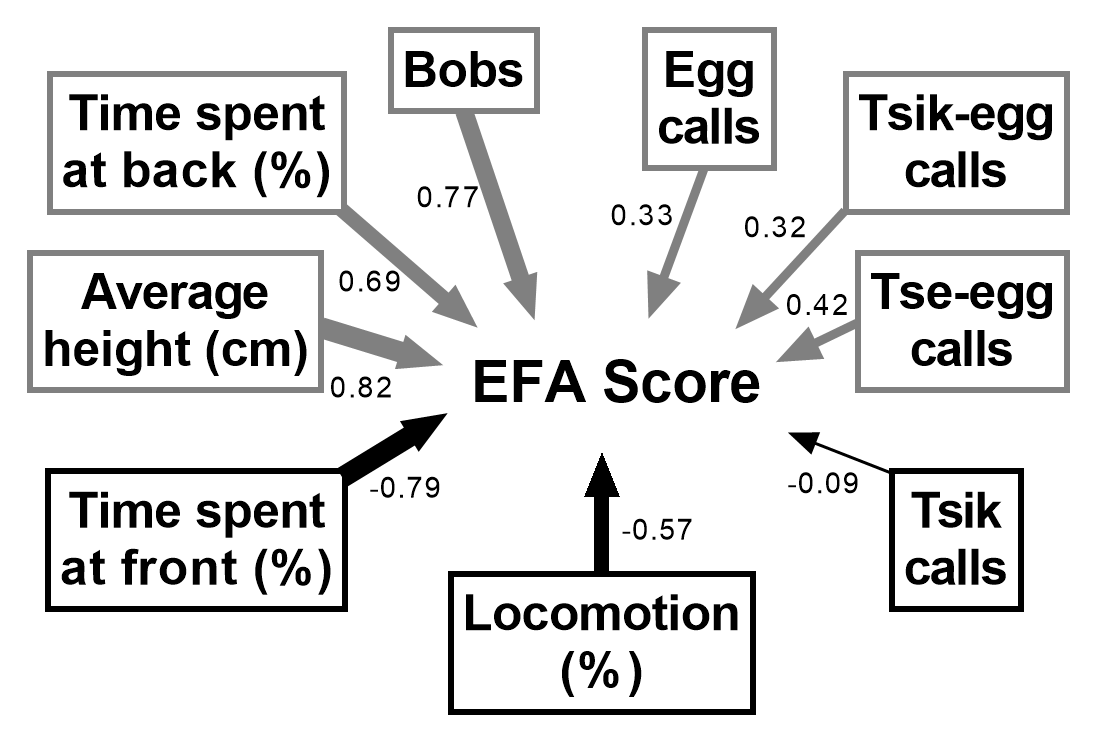


**Fig S7. Weightings of behavioural variables that contribute to the single latent factor derived from the exploratory factor analysis (EFA) in the human intruder test.** Behaviour and vocalisations that contribute to the multifaceted threat response towards the human intruder were assessed in Quah et al., (*49*) in an exploratory factor analysis of 171 marmosets. A single latent factor explained the data. Variables boxed in grey weight positively on the factor, increasing the overall score and indicate a heightened avoidance response to the human intruder. Variables boxed in black weight negatively on the factor, with increases in these variables decreasing the score and indicate a reduced avoidance response to the human intruder. Weightings for each variable are located next to each arrow.


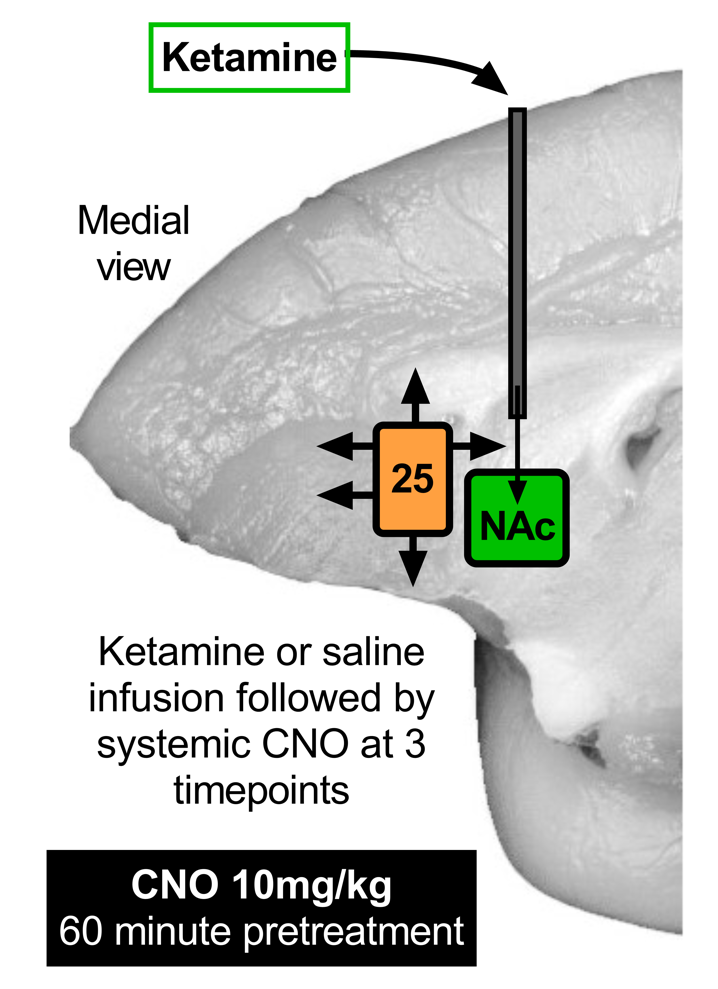


**Fig S8. Schematic of scACC-25 activation following intra NAc ketamine infusion**. Ketamine (0.5µg; or saline as a control) was infused into the NAc through intracerebral cannulae. Following this 10mg/kg CNO was administered systemically at 3 timepoints post-infusion to induce blunting of anticipatory arousal in the appetitive Pavlovian discrimination test.


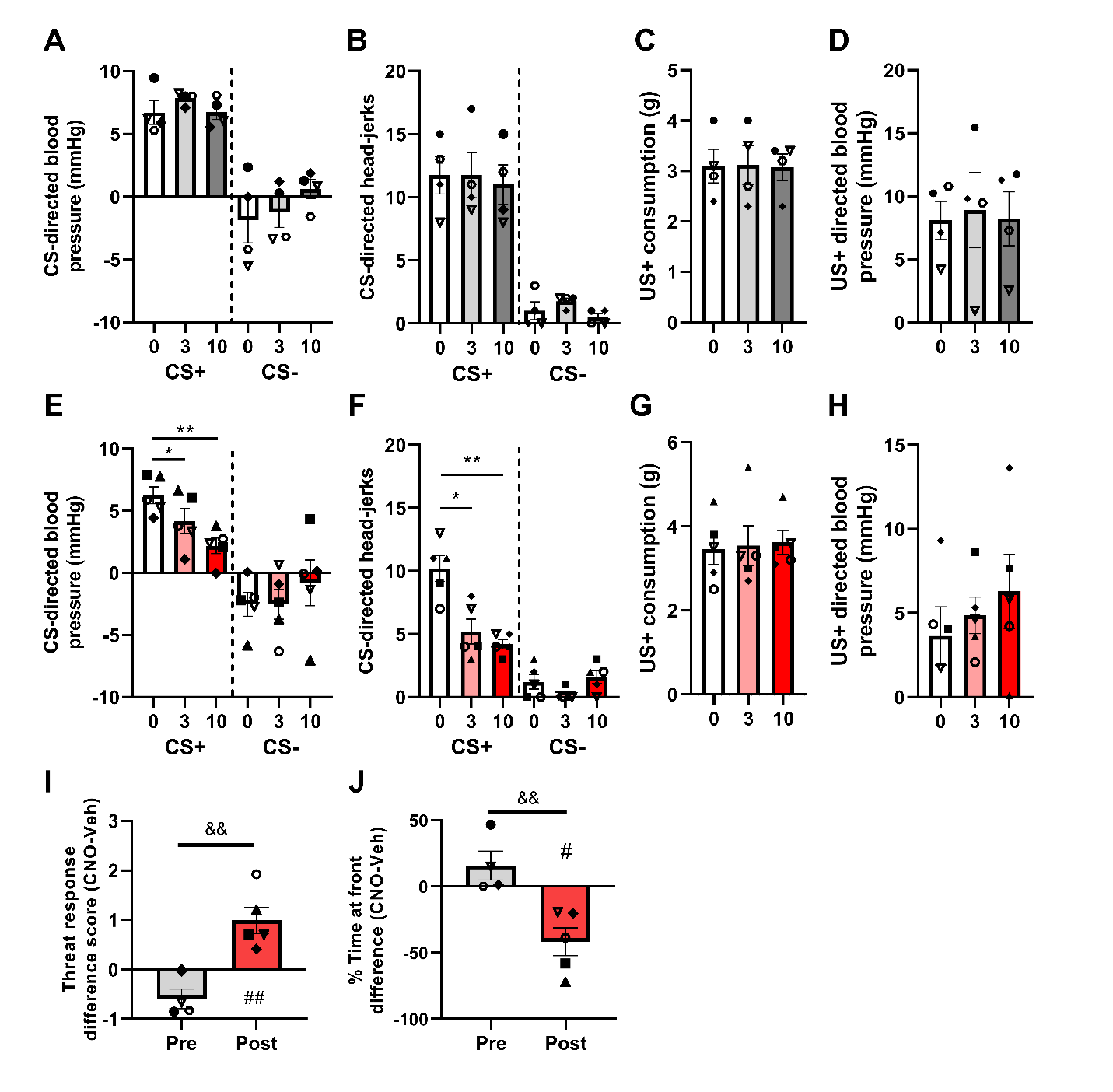


**Fig S9. Validation of DREADD-specific effects of CNO treatment on scACC-25 induced blunting of anticipatory arousal and heightening of uncertain threat reactivity.** (**A**) Before DREADD infusion (n=4), CNO (3 or 10 mg/kg) compared to vehicle ‘0’ did not effect CS+ or CS- directed blood pressure responses (two-way rmANOVA, treatment*CS, F_(1.2,3.6)_=4.1, p=0.12). (**B**) Equally, CNO did not influence head-jerk behaviour prior to DREADDs infusion (two-way rmANOVA; treatment*CS, F_(2,6)_<1). (**C**) Similarly, consumption of the marshmallow reward (US+) was unaffected by CNO treatment (rmANOVA, treatment, F<1), and (**D**) US+ blood pressure responses were also unaffected (rmANOVA, treatment, F<1). (**E**) Post-DREADDs infusion (n=5), CNO treatment blunted the anticipatory CS+ directed blood pressure responses at both doses (two-way rmANOVA, treatment*CS interaction, F_(1.9,7.4)_=5.4, p=0.037, η^2^=0.58; Sidak-corrected posthoc: Veh vs 3, p=0.013, *d*=1.1; Veh vs 10, p=0.004, *d*=2.8). (**F**) Equally, post-DREADD CNO treatment blunted the anticipatory behavioural response to the CS+ at both doses (two-way rmANOVA, treatment*CS, F_(2,8)_=46, p<0.001, η^2^=0.92; Sidak-corrected posthoc: Veh vs 3, p=0.019, *d*=2.2; Veh vs 10, p=0.006, *d*=3.5). (**G**) The DREADD-mediated anticipatory effects did not extend to consummatory responses with US+ consumption unaffected by CNO treatment (rmANOVA, treatment, F<1), (**H**) with US+ blood pressure responses also unaffected (rmANOVA, treatment, F_(1.3,5.2)_=1.3, p=0.32). (**I**) In the human intruder paradigm, CNO treatment increased the threat response difference score (CNO-Vehicle) in the Post-DREADD group (one sampled t-test vs hypothetical mean of 0, p=0.02, *d*=1.6) but not Pre-DREADD group (one samples t-test vs hypothetical mean of 0, p=0.057). This DREADD-mediated CNO effect was also reflected by the significant Pre- vs Post-DREADD comparison (unpaired t-test with Welch’s correction, p=0.002, *d*=3.1). (**J**) Contributing to the Post-DREADD heightened threat response was a decrease in time spent at the front of the cage following CNO treatment (one samples t-test vs hypothetical mean of 0, p=0.016, *d*=1.8), which was not observed in the Pre-DREADD group (one sample t-test vs hypothetical mean of 0, p=0.24). Comparison between these groups further illustrates this DREADD-mediated effect (unpaired t-test with Welch’s correction, p=0.0069, *d*=2.6). All data are displayed as means with SEM error bars and individual data points for each subject. Significance values are indicated as p<0.05*, p<0.01**. Within subject comparisons are denoted by *, between subject comparison by & and one sampled t-test versus a hypothetical mean of 0 by #. Histogram bars in red denote CNO treatment post DREADDs infusion whilst histogram bars in grey denote CNO treatment pre DREADDs infusion.

**
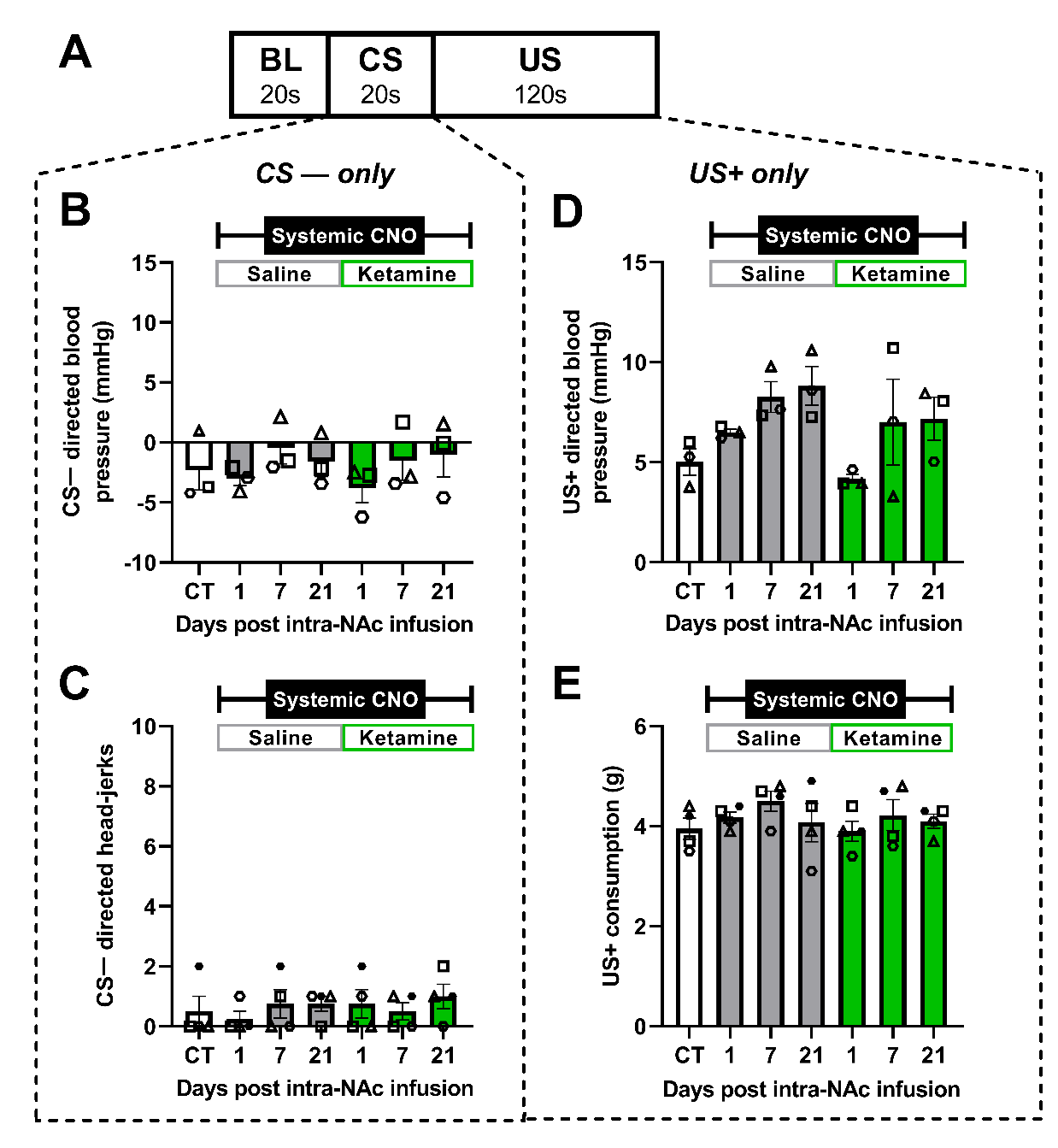
**

**Fig S10. Intra-accumbens ketamine and systemic CNO treatment do not affect arousal during CS- trials or consummatory responses.** (**A**) Trial schematic indicating length of each component with data from the CS- periods provided. These periods are paired with an empty foodbox (US-) so do not generate the anticipatory cardiovascular or behavioural response, as can be seen from the control data point (CT) prior to this drug study. (**B**) Infusion of ketamine or saline prior to systemic CNO treatment (10mg/kg) did not impact on CS- directed MAP responses at the 3 different time points (n=3, two-way rmANOVA, treatment*time F<1, p=0.649), with minimal MAP response across all groups. (**C**) Similarly, neither infusion impacted the effects of systemic CNO treatment on CS- directed head-jerk behaviour (n=4, two-way rmANOVA, treatment*time F<1, p=0.706), with minimal head-jerk behaviours across all groups. Data are displayed as means with SEM error bars and individual data points for each subject. (**D**) The US+ MAP response was also unaffected by systemic CNO treatment at these different time points post ketamine or saline infusion (n=3, two-way rmANOVA, treatment*time, F<1, p=0.903). Note that the cardiovascular probe of subject 3 stopped working so only behavioural responses are shown. (**E**) Systemic CNO treatment (10mg/kg) at 1, 7 and 21 days post ketamine or saline infusion did not influence US+ consumption (n=4, two-way rmANOVA, treatment*time, F<1, p=0.734).

Supplementary Tables

| **Measure** | **EFA**  **contribution** | **Nucleus Accumbens** | | **Amygdala** | | **Test statistic** | |
| --- | --- | --- | --- | --- | --- | --- | --- |
|  |  | **Veh** | **CNO** | **Veh** | **CNO** | **F / X2** | **p** |
| **Time spent at front, %** | -0.790 | 33.2 (8.39) | 40.5 (12.5) | 39.4 (5.50) | 21.8 (2.43) | 4.816 | 0.093 |
| **Time spent at back, %** | 0.688 | 23.1 (5.21) | 24.7 (8.89) | 16.8 (5.42) | 36.9 (2.96) | 6.796 | 0.06 |
| **Height, cm** | 0.816 | 47.3 (7.09) | 45.4 (2.34) | 41.1 (5.10) | 50.3 (3.91) | 1.974 | 0.233 |
| **Locomotion, %** | -0.568 | 8.76 (1.54) | 7.97 (1.33) | 10.2 (0.89) | 9.37 (1.15) | 0 | 0.998 |
| **Head and body bobs** | 0.769 | 5.8 (3.4) | 7 (4.06) | 5.8 (1.32) | 5.2 (2.22) | 2.125† | 0.547 |
| **Tsik calls** | -0.091 | 0 | 1.4 (0.68) | 0.4 (0.25) | 0 | 6.556† | 0.087 |
| **Tsik-egg calls** | 0.323 | 12.8 (10.7) | 5.8 (5.55) | 4 (2.10) | 0.4 (0.4) | 3.923† | 0.27 |
| **Tse-egg calls** | 0.417 | 5.2 (3.56) | 4.8 (3.09) | 3.6 (2.66) | 2.6 (2.14) | 1.345† | 0.719 |
| **Egg calls** | 0.332 | 2.8 (2.33) | 3 (2.28) | 4.2 (1.69) | 4.6 (2.02) | 0.907† | 0.824 |
| **Tse calls** |  | 0 | 1 (1) | 0 | 0 | 3† | 0.392 |
| **Jumps** |  | 1.4 (0.51) | 0.4 (0.25) | 0.8 (0.37) | 0.4 (0.25) | 3.469† | 0.325 |

**Table S1. Heightened threat response as reflected by increased score on EFA-derived single latent factor, induced by selective activation of scACC-25-to-amygdala pathway, did not affect any individual measure.** No individual measure was altered following scACC-25 pathway activation by CNO in either region (n=5), although when amalgamated to create the threat response score, the latter did show an overall increase following activation of the amygdala pathway (Fig 3B in main manuscript). This reflects the fact that a range of behaviors displayed towards the human intruder contribute to the exploratory factor analysis (EFA)-derived threat response score, but individual marmosets display distinct patterns of these behaviors. It can be seen though that there were trends toward an interaction between treatment and region for the time spent at the front and back of the cage, with lowered time spent at the front following CNO in the amygdala and heightened time spent at the back. This suggests that after CNO infusion into the amygdala the marmosets, in general, avoided the human intruder. Test statistics indicate treatment*interaction output values following a two-way ANOVA (parametric data) or Freidman test (non-parametric data), with † indicating the latter. Mean vehicle and CNO data are provided for both nucleus accumbens and amygdala targeted infusions, with the standard error of the mean in brackets.

| **Subject/ Symbol** | **Sex** | **scACC-25 pathway activation** | | **Central Ketamine study** | **Systemic DREADDs validation** | |
| --- | --- | --- | --- | --- | --- | --- |
|  |  | **Appetitive** | **Distal threat** |  | **Pre-DREADDs** | **Post-DREADDs** |
| Main study | | | | | | |
| 1 △ | F | ✓ | ✓ | ✓ |  |  |
| 2 □ | M | ✓ | ✓ | ✓ |  |  |
| 3 ⬢ | F | ✓^†^ | ✓ | ✓^†^ |  |  |
| 4 ◇ | M |  | ✓ |  |  |  |
| 5 ⬡ | M | ✓ | ✓ | ✓ | ✓ |  |
| Supplementary data | | | | | | |
| 6 ⚫ | F |  |  |  | ✓ |  |
| 7 ▽ | F |  |  |  | ✓ | ✓ |
| 8 ◆ | M |  |  |  | ✓ | ✓ |
| 9 ▲ | F |  |  |  |  | ✓ |
| 10 ⭘ | F |  |  |  |  | ✓ |
| 11 ◼ | M |  |  |  |  | ✓ |

**Table S2. Subject involvement across the main study and supplementary data.** A tick indicates subject participation in the distinct phases of each study. A dagger symbol indicates the subject’s cardiovascular data was unusable due to a communication failure within the implanted telemeter.
